# How to incorporate the dose-rate effect into evaluation of cancer risk for radiation protection

**DOI:** 10.1093/jrr/rru009

**Published:** 2014-03-23

**Authors:** Kouichi Tatsumi, Hiroshi Tanooka

**Affiliations:** 1 Health Research Foundation, 103-5 Tanaka-Monzen-cho, Sakyo-ku, Kyoto 606-8225, Japan; 2 National Cancer Center Research Institute, Tsukiji, Chuo-ku, Tokyo 104, Japan; 3 Radiation Effects Association, 1-9-16 Kaji-cho, Chiyoda-ku, Tokyo, Japan

The current radiation rules, based on epidemiological data obtained from the cancer risk of A-bomb survivors, were formulated in the context of extremely high dose rates. The International Commission for Radiological Protection (ICRP) proposed a dose and dose-rate effectiveness factor (DDREF) of 2 based on two high and low dose-rate categories of A-bomb data for an extremely high dose-rate range (ICRP Publication 60, 1991). From a scientific point of view, this value is valid only for the A-bomb dose-rate range, i.e. an extremely high dose rate, and cannot be applied at an environmental level. Nevertheless, this dose-rate factor is currently widely applied in estimating the cancer risk of low dose and low dose-rate radiation and creates an overestimation of cancer risk from radiation at the environmental level, such as in the Fukushima region following the accident at the nuclear power plant. Overestimation of radiation risk results in unnecessary psychological and economical burden on our society. There is a need to reassess the validity of the currently adopted understanding of the dose-rate effect of radiation on cancer risk.

This paper is a report of a symposium organized for that purpose under the sponsorship of the Health Research Foundation, Kyoto, at the 56th Annual Meeting of the Japan Radiation Research Society, held in Aomori, Japan, on 18 October 2013. The program for the symposium included the following presentations.

- Basis of the dose-rate effect of radiation (Hiroshi Utsumi, Health Research Foundation)
- Effects of long-term exposure to low dose-rate radiation on mice (Tetsuya Ono, Institute for Environmental Sciences)
- Effects of Chernobyl accidents on mental health and their lesson in setting radiation protection policy after the nuclear accident in Japan (Keishiro Ito, formerly with the Institute for Policy Sciences)
- Theory and data for estimation of risk from low dose and low dose-rate radiation (Michiaki Kai, Oita University of Nursing and Health Sciences)

Co-chairs: Kouichi Tatsumi (Health Research Foundation) and Hiroshi Tanooka (Radiation Effects Association)

Utsumi discussed the fidelity of the DNA repair mechanism and its dependence on the dose rate of radiation. After induction of double-strand breaks in DNA by radiation, the broken ends undergo two repair processes in cells, i.e. homologous end joining and non-homologous end joining. Homologous end joining has been thought to be error free and non-homologous end joining to be error prone. However, recent findings show that the major part of non-homologous end joining is also error free. From these observations, the DNA repair mechanism seems to be prone to fewer errors than previously thought. Since it is assumed that one DNA double-strand break per cell per day allows cells to survive error free, and the dose rate of radiation needed to cause this break is 33.3 mSv/d, i.e. 2.3 × 10^−5^ Sv/min, Utsumi suggested that this is a tolerable dose rate for life under continuous exposure to low-LET radiation from the point of view of the DNA repair mechanism.

Ono summarized up-to-date data from experiments being conducted at the Institute for Environmental Sciences (IES). A group of 500 mice each were exposed continuously to γ radiation from ^137^Cs for 400 days at various dose rates, including 0.05, 1 and 20 mGy/d (22 h exposure per day). These dose rates are about 20-, 400-, and 8000-fold higher than the natural radiation background level. In the 0.05 mGy/d and 1 mGy/d groups (total doses received: 0.02 and 0.4 Gy, respectively), the mice showed no effect from the radiation on the cancer incidence rate or chromosome aberration, although a slight increase in mRNA expression was detected in three genes. In the 20 mGy/d group (total dose, 8 Gy), the mice showed a considerable increase in the cancer incidence rate. Even so, the increase was 2–3-fold lower when compared with cancer data obtained with the higher dose rate of 1 Gy/min on the same strain of mice (Sasaki *et al.*). Furthermore, a large discrepancy between these cancer incidence rates (as observed at IES) and the cancer incidence rate estimated by extrapolation from A-bomb data by applying the linear non-threshold (LNT) model (0.5% cancer increase by 100 mSv) was pointed out at the discussion. Whether this discrepancy is due to a difference in radiosensitivity between humans and mice or to the difference in the dose-rate effect awaits further analysis. In other experiments, the frequency of chromosome translocations in the mouse spleen was drastically decreased by lowering the dose rate from 890 to 0.3 mGy/min. However, this frequency remained unchanged by lowering the dose rate further. Ono noted the presence of some effects on health due to radiation exposure at the space station, which did not occur at lower dose rates.

Ito discussed the psychological and socio-economic problems in setting the radiation protection regulation. He introduced the WHO report (2006) that described the mental health effect of the Chernobyl nuclear accident as important as the visible effects such as cancer. The possibility that the radiation protection regulation rule itself creates similar problems has been discussed from the point of view of regulatory impact analysis. Nevertheless, in 2008 the ICRP recommended a severe regulation level of 1 mSv/year (Publication 111). Application of these radiation protection rules created a considerable psychological and socio-economic impact after the nuclear accident in Fukushima. It seems that the Chernobyl lesson was not well incorporated in setting the Japanese regulatory rules. Application of a severe radiation protection rule causes unnecessary psychological stress on people. Ito concluded that attention should be paid to the psychological and socio-economic effects in setting the radiation protection and evacuation rules post-Fukushima.

Kai explained the theory and the method of evaluating risk from radiation exposure in response to the discussion on the dose-rate problem. The current method for evaluation of the cancer risk of radiation is based on data from epidemiological study of A-bomb survivors. However, a discrepancy between that data and biological research observations has remained unexplained. Although various models have been tested, the most appropriate model for estimation of the radiation cancer risk is the LNT model, as approved by ICRP, the National Academy of Sciences USA, and the United Nations Scientific Committee. Currently, a value of 2 or less for the DDREF is used for estimation of cancer risk from low dose and low dose-rate radiation. Its applicability is now being revised in a task group of the ICRP.

The organizers believe that the dose-rate problem is an important and urgent issue for our society to consider, not only from a scientific aspect but also from a social aspect. We hope to discuss this problem further and thus to propose an appropriate value for the DDREF in the future.

